# Mechanical Characterization of the Plastic Material GF-PA6 Manufactured Using FDM Technology for a Compression Uniaxial Stress Field via an Experimental and Numerical Analysis

**DOI:** 10.3390/polym12010246

**Published:** 2020-01-20

**Authors:** Jorge Manuel Mercado-Colmenero, Cristina Martin-Doñate, Vincenzo Moramarco, Michele Angelo Attolico, Gilda Renna, Moises Rodriguez-Santiago, Caterina Casavola

**Affiliations:** 1 Department of Engineering Graphics Design and Projects, University of Jaen, Campus Las Lagunillas, s/n. Building A3, 23071 Jaen, Spain; jmercado@ujaen.es (J.M.M.-C.); mrs00025@red.ujaen.es (M.R.-S.); 2 Dipartimento di Meccanica, Matematica e Management (DMMM), Politecnico di Bari, Viale Japigia, 182–70126 Bari, Italy; vincenzo.moramarco@poliba.it (V.M.); micheleangelo.attolico@poliba.it (M.A.A.); gilda.renna@poliba.it (G.R.); caterina.casavola@poliba.it (C.C.)

**Keywords:** additive manufacturing, plastic part design, CAD, FDM, numerical simulation, experimental tests

## Abstract

This manuscript presents an experimental and numerical analysis of the mechanical structural behavior of Nylstrong GF-PA6, a plastic material manufactured using FDM (fused deposition modeling) technology for a compression uniaxial stress field. Firstly, an experimental test using several test specimens fabricated in the Z and X-axis allows characterizing the elastic behavior of the reinforced GF-PA6 according to the ISO 604 standard for uniaxial compression stress environments in both Z and X manufacturing orientations. In a second stage, an experimental test analyzes the structural behavior of an industrial part manufactured under the same conditions as the test specimens. The experimental results for the test specimens manufactured in the Z and X-axis present differences in the stress-strain curve. Z-axis printed elements present a purely linear elastic behavior and lower structural integrity, while X-axis printed elements present a nonlinear elastic behavior typical of plastic and foam materials. In order to validate the experimental results, numerical analysis for an industrial part is carried out, defining the material GF-PA6 as elastic and isotropic with constant Young’s compression modulus according to ISO standard 604. Simulations and experimental tests show good accuracy, obtaining errors of 0.91% on the Z axis and 0.56% on the X-axis between virtual and physical models.

## 1. Introduction

Additive manufacturing (AM), also recognized as 3D printing, is a technological system used in the fabrication of 3D components from CAD designs. The additive manufacturing technology diverges basically from subtractive manufacturing technologies such as CNC in that the element to be fabricated is achieved from the progressive addition of material in layers, which moderates manufacturing refuse [1,2]. This layer-by-layer process allows for great freedom in manufacturing free form geometries in plastic that cannot be produced through standard manufacturing technologies, such as for example injection molding [3,4,5,6,7,8,9]. There are several processes established based on additive manufacturing technology: stereolithography, selective laser sintering, molten deposition modeling (FDM), etc. In this area, fused deposition modeling (FDM) is the most commonly employed for its simple use, low cost and high efficiency [10]. In the FDM technology, a filament of plastic material is inserted into the extruder at the suitable pressure. The positioning of the extruder by a CNC-based code attached to the extrusion process of a semi-molten filament above the material’s glass transition temperature allows the manufacturing of the designed topology [11]. Currently, the FDM manufacturing process has grown from a prototyping method to manufacturing technology, transforming prototypes into finished end parts [12,13]. The production process with FDM technology is especially encouraging in the fabrication of end parts or low-volume products. This tendency emphasizes the demand for a full knowledge of the mechanical specifications of parts obtained by layer deposition procedures. Not only should the plastic material be resistant, but also the mechanical specifications of the layered elements must meet the operational product specifications.

In the FDM manufacturing process, thermoplastic materials such as PC, ABS, and PLA are mainly used due to their low melting temperature. However, most of these polymers present average values of mechanical strength according to the product requirements. This disadvantage restricts the application of FDM for industrial products. One way to improve the mechanical behavior in the FDM process is the use of composite filaments. These filaments are basically composed of a polymer matrix with reinforcing fibers. In this way, it is possible to improve the mechanical and structural properties of the final polymer over its individual components [10]. As fibrous reinforcements, short fibers [14,15,16], fibrils [17], nanofibers [18] and continuous fibers [19,20,21] have been the most utilized. The fibers are usually combined with thermoplastic matrix materials such as Nylon, PLA, ABS, PPS, and PEI. Nylon or polyamide is a biocompatible polymer with good mechanical properties and excellent processability in FDM manufacturing. Polyamide is currently used in automotive parts [22] and in medical components [23]. In the area of polymers, polyamide 6 (PA6) is considered very suitable as a thermoplastic matrix, mainly due to its good thermal stability, its low dielectric constant and its great strength, as well as its low cost.

Unfortunately, and despite the advantages of filaments made of composite materials, the properties of the components manufactured with FDM depend not only on the type of material and fiber but also on other factors related to the manufacturing process and product design. The part building axis, bead width, air pockets, raster angle, layer thickness, number of contours and temperature are the most significant aspects in the FDM technology. Particularly, the FDM production process is identified by an anisotropic behavior, caused by the directional material deposition. Many authors have centered their work on obtaining the influence of the manufacturing parameters on the mechanical requirements of FDM parts [24,25,26,27,28,29,30,31,32,33,34]. Croccolo et al. [24] presented the influence of the FDM manufacturing specifications on the tensile strength and on the stiffness of the parts manufactured with ABS-M30, undertaking the problem from an experimental and analytical approach. Tanikella et al. [25] performed the mechanical specifications of FDM parts employing a commercial 3D printer for a wide variety of materials, testing the samples for tensile strength. Casavola et al. [26] characterized test specimens produced by FDM by using the classical laminate theory, in [27] measuring the residual stress by means of the hole drilling measure procedure and in [28] characterizing the mechanical behavior of FDM components under impact tensile loadings. Li et al. [29] presented quantitative research into the warping of PLA printed parts according to the interface residual stress. Chacon et al. [30] studied the problem of build orientation, layer thickness and feed rate on the mechanical specifications of PLA test specimens produced with a standard 3D printer. Kuznetsov et al. [31] presented the effect of geometrical requirements of FDM on manufactured part strength for PLA material and standard 3D printers. In [32] they performed several samples of printed parts with interrupted shells, studying the impact of shell and thicknesses including the infill density on the piece strength. Puigoriol-Forcada et al. [33] showed the effect of the building orientation in the manufacturing of the PC printed parts subjected to flexural fatigue loads. Torres et al. [34] examined the effects of main manufacturing specifications on the final material behavior of FDM PLA elements subjected to torsion loads.

An industrial FDM printed part requires high quality and minimum anisotropy. FDM manufactured elements should align the load/stresses with the strongest orientation of the material. According to this fact, plastic polymers have better mechanical characteristics in compression stresses than in tensile stresses. This situation is important when manufacturing components for mechanical applications or industrial products produced with the FDM process. Some authors have analyzed the mechanical compression properties of polymers produced with FDM by means of experimental analysis. Upadhyay et al. [35] studied the influence of build position on the mechanical features of ABS P400 test specimens, studying tensile and compressive strength, Izod impact and hardness. Lee et al. [36] presented the results of the anisotropic compressive strength of FDM produced parts. Sood et al. [37] performed an analysis to study the influence of five requirements in 3D printing such as layer thickness, part build orientation, raster angle, raster width and air gap of test specimens on a compressive stress field. Using the PA 6 material, Miguel et al. [38] analyzed and evaluated the water absorption, mechanical properties and consequent failure surfaces of nylon parts obtained by FDM, with two raster angles, after being coated with protective material. The performance of the coated parts was evaluated with water absorption tests, as well as compression and tensile tests.

The development of composite materials specifically for AM enhances the mechanical plastic properties over existing polymeric materials [39]. In this line, Brenken et al. [40] employed the FDM process to fabricate continuous carbon and glass FRTP composites in order to analyze the tensile, flexural, and quasi-static indentation characteristics of the printed composites. Ngo et al. [41] modified ABS by incorporating short glass fibers, obtaining as a result that glass fibers improved the strength of an ABS filament by reducing its flexibility. 

New manufacturing processes like the FDM process have raised new design needs to resolve. One of these needs is the simulation of plastic parts with functional requirements, designed to be manufactured using the FDM process. Undertaking a new design concept requires the validation of its mechanical performance with FEM simulations before its manufacture. The mechanical characterization of materials for technological processes such as injection molding is not valid for the numerical simulation of additively manufactured parts using the FDM process. For industrial parts produced with the FDM process, the material specifications vary particularly with respect to those of a uniform material whose technical requirements are clearly detailed in the technical datasheets. Numerical simulation needs a precise understanding of the mechanical material specifications; these being affected by the fabricating technology as well as by the stresses to which the product will be exposed in their working positions. Considering that these specifications are not usually achievable, it is recommended to perform several experimental tests before the simulation by employing test specimens produced using the same specifications as the tested part. An experimental test normally expends a lot of time and money. In addition, and in order to finish the part validation from the experimental results, it is necessary to recognize the best way to use the experimental results in the FEM software in order to obtain simulation analyses that correspond with the experimental material performance. All these reasons mean that the industrial part design of pieces manufactured with FDM can still be a complex engineering problem. Given the enormous importance of properly characterizing materials in the field of numerical simulation, unfortunately, insufficient attention has been paid to the mechanical characterization of the FDM process using individual or composite plastic materials. This fact causes a serious problem among engineering design professionals because they require this information to accurately simulate the behavior of components manufactured with FDM technology [8]. Finite element analysis is not able to accurately validate the mechanical behavior of parts manufactured with FDM technology in the same way that it predicts the behavior of isotropic parts manufactured with traditional methods. In addition, the difficulty increases as there are currently very few research studies that validate the material features in terms of its mechanical strength. It is possible to use approximate information in the finite element software to numerically simulate the behavior of the printed part with FDM, however, the results will not be reliable for secure validation of the component. In order to use and produce parts manufactured with FDM at an industrial level, it is necessary to know exactly their mechanical properties [42]. The use of simulation tools to validate parts manufactured with FDM and model the production process is currently in its beginnings [43].

Many research papers study the material characterization and the mechanical specifications of materials for FDM based on the information obtained from experimental tests only for test specimens. For the specific case of PA6 reinforced with glass fiber subject to uniaxial compression tests, there is no research or industrial work that characterizes the material according to the FDM technology [44]. For real industrial uses, it is important to know whether the material’s mechanical properties could be employed for predicting the mechanical behavior of FDM end parts. According to [44] there are very few scientific studies on this area as this subject is contemplated as a new research line.

In order to solve these problems, this paper presents an experimental and numerical investigation of the mechanical structural behavior of the Nylstrong GF-PA6 plastic material manufactured using FDM technology for a compression uniaxial stress field. Additionally, the research presented here validates the characterization of the reinforced GF-PA6 material employing a 3D printed industrial part. These results allow us to reach conclusions that very few research papers can since most of the research presented shows only general results on test specimens [45]. The structural behavior of the real industrial part is also validated according to the design requirements of the assembly in which it is located. The results of our research indicate that the employment of GF-PA6 material is promising in FDM manufacturing processes to obtain industrial parts with low production volume subjected to compression efforts or for real parts manufactured by the user.

## 2. Materials and Methods

### 2.1. Geometrical and Functional Specifications for the Study Part Manufactured with the FDM Process

**Figure 1:**
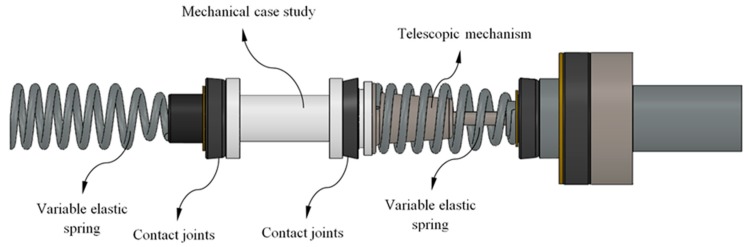
Automotive assembly.

Below we present the geometrical requirements of the FDM mechanical part under study. In addition, aspects that affect the selection of the 3D FDM material in the boundary mechanical specifications and in the load scenario for the studied part are described. The mechanical part which is the object of study belongs to an automotive system shown in Figure 1.

The end part under study presents a cylindrical geometry of revolution with a variable cross-section along its longitudinal axis (see Figure 1). This part acts as a centering axis for an elastic spring and a telescopic mechanism with an elastic and damping component. Both elements, spring and mechanism, are located on the two ends of the part. The links between elements that make up the automotive assembly (see Figure 1) are represented by contacts activated by the loads supported by the printed part. The study part has been produced with an FDM additive process and by using a reinforced plastic material composed of a PA6 matrix, which is Nylon, and fiberglass. The reinforced GF-PA6 has been adapted and optimized by the supplier of the material [46] for use in the FDM process. The commercial name for the reinforced GF–PA6 [47] is Nylstrong. GF–PA6 is characterized by having a great impact strength and an appropriate balance between hardness and mechanical strength. Additionally, its high thermal strength makes GF–PA6 suitable for industrial and mechanical purposes [47]. The GF–PA6 filament comprises polyamide 6 with fiberglass, polyamide 6 with glass spheres and calcium carbonate as nucleant, with the ratio between spheres and fibers being 20%–30% [47]. A disadvantage in using Nylon PA6 for manufacturing industrial applications is the fact that this material absorbs moisture, reducing the strength and stiffness of the part obtained. To solve this problem, the GF–PA6 filament prior to the manufacturing process should undergo an isothermal heating treatment based on drying the filament in an oven at 60 °C for a period of approximately 6 h. Table 1 shows the physical, mechanical and main Nylstrong GF–PA6 characteristics for FDM uses [47].

The part under study is subjected to a uniaxial compression stress state, determined by the contact of the elastic components that make up the mechanical assembly (see Figure 1). The load scenario is defined by a unidirectional axial force applied at one end of the part, the boundary condition being a fixed support at the opposite end. The compression tensile yield stress for the printed end part has been obtained analytically from the classical structural analysis, introducing the values of the uniaxial compression load to which the part is subjected and the tensile yield stress of Nylstrong GF–PA6 (see Equation (1)).

(1) $$\sigma_{y}=\int_{L_{o}}^{L}\frac{F_{c}}{A_{\mathrm{cross}}\left(z\right)}\mathrm{dz}$$

**Figure 2:**
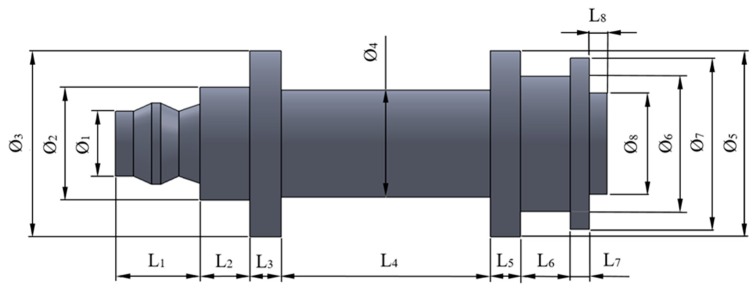
Topology of the studied end part.

**Figure 3:**
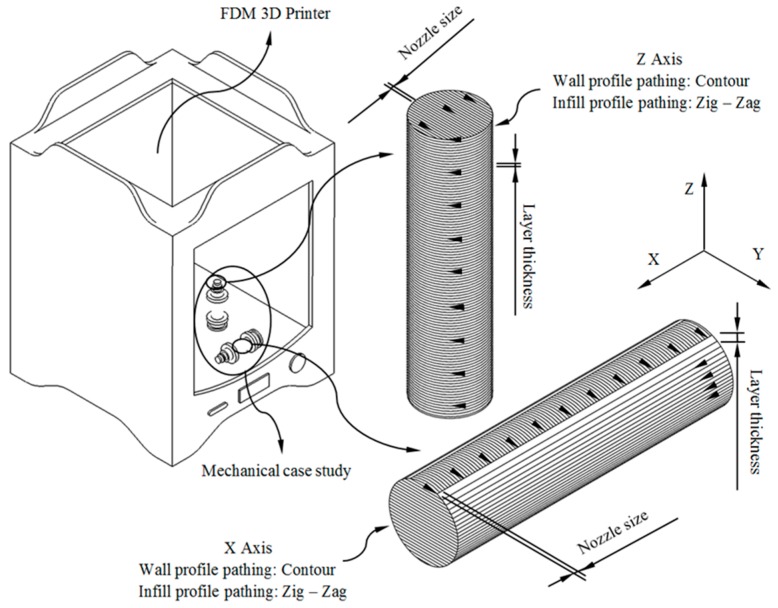
FDM (fused deposition modeling) process configuration for the manufacture of the end part.

The tensile yield stress of the studied end part is given by σ_y_ [MPa], *A*_cross_ [mm^2^] is the area of the part cross-section, *F*_c_ [N] is the uniaxial compression load and *L* represents the part length along the Z axis (see Figure 2 and Figure 3, and Equation (2)).

(2) $$L=L_{1}+L_{2}+L_{3}+L_{4}+L_{5}+L_{6}+L_{7}+L_{8}$$

The global dimensions of the mechanical part have been determined by the requirements and specifications of the final product design. The variables *F*_1_, *F*_2_, *F*_3_, *F*_4_, *F*_5_, *F*_6_, *F*_7_, and *F*_8_ indicate the dimensions of the different diameters and *L*_1_, *L*_2_, *L*_3_, *L*_4_, *L*_5_, *L*_6_, *L*_7_, and *L*_8_ indicate the set of partial lengths along the axis. The values of the set of variables are presented in Table 2.

The manufacture of the part has been carried out via FDM technology using the 3D printer Ultimaker 2+ [48]. The printing dimensions of the FDM printer are 223 mm on the X-axis, 223 mm on the Y-axis and 205 mm on the Z axis. The mechanical properties of additive manufactured parts are affected by both the unprinted material properties and the manufacturing method. FDM manufactured parts for industrial applications must be structurally resistant in a similar way to the original part produced with conventional processes (for example, injection molding). It is recommended when possible to align the load/stresses with the strongest orientation of the fiber by means of the manufacturing direction of the component. In order to analyze the manufacturing direction that maximizes the structural behavior and compressive strength of the manufactured parts made of GF–PA6, six (Z-axis, see Figure 3) and five (X-axis, see Figure 3) test specimens have been manufactured. The geometries presented have identical dimensions, with five of them manufactured in a horizontal position along the transverse manufacturing direction (X-axis) and the rest in a vertical position along the Z axis (see Figure 3). The specimens have been manufactured from top to bottom following the positive axis direction of the FDM printer. Thus it is possible to analyze the influence on the mechanical behavior of the different manufacturing orientations in a scenario of stress in uniaxial compression. This information is relevant for the real industrial manufacture of the part.

As shown in Figure 3, the geometrical paths used to manufacture the layers of the printed parts have been contour profiles for the external wall and zig-zag paths for the inlet pattern. Table 3 indicates the values of the main parameters used for configuring the production process. The product requirements for the printed part have been adjusted to the specifications demanded for the FDM manufacturing process. The printed parts required supports for their manufacture both in horizontal positioning along the X-axis and vertical positioning along the Z-axis.

**Figure 4:**
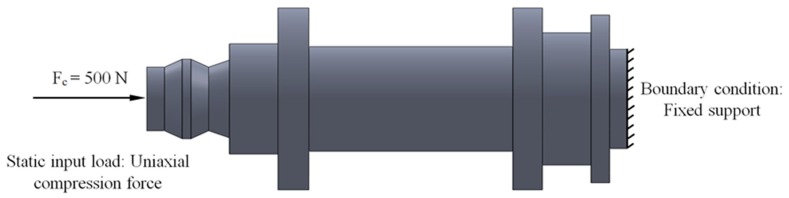
Requirements and load definition for the printed part.

The set of loads and the boundary specifications for the printed part are presented in Figure 4. As shown, the maximum compressive force that the part has to bear in working position is equal to 500 N. This force is applied to the opposite end of the part to which the boundary requirements have been proposed (see Figure 4). The boundary specification established for the studied part is a fixed support on the opposite side to where the force is applied (see Figure 4).

### 2.2. Experimental Tests

In the present manuscript, two experimental analyses have been carried out in order to evaluate the structural behavior of the printed part and mechanically characterize the behavior of the reinforced material Nylstrong GF–PA6. Firstly, the mechanical and elastic features of Nylstrong GF–PA6 have been characterized by using 3D printing specimens with prismatic geometry following the requirements of the ISO-604 standard (2003) [49]. Secondly, the printed part under study was subjected to an experimental test to evaluate its structural behavior under a uniaxial compression stress state. (see Figure 4). The results of the experimental tests allow us to establish mechanical parameters such as compression yield stress, displacements and loads along its elastic area until reaching the compression yield stress, compressive stiffness, and ultimate yield stress. Successively, after the mechanical and elastic characterization of the Nylstrong GF–PA6 a set of static structural numerical simulations has been defined for the 3D printed part. In this line, it is possible to contrast the results obtained from the numerical simulations with the results obtained in the experimental tests, validating the methodology for defining and configuring the numerical simulations and characterizing the elastic and mechanical behavior of the reinforced plastic material under study.

**Figure 5:**
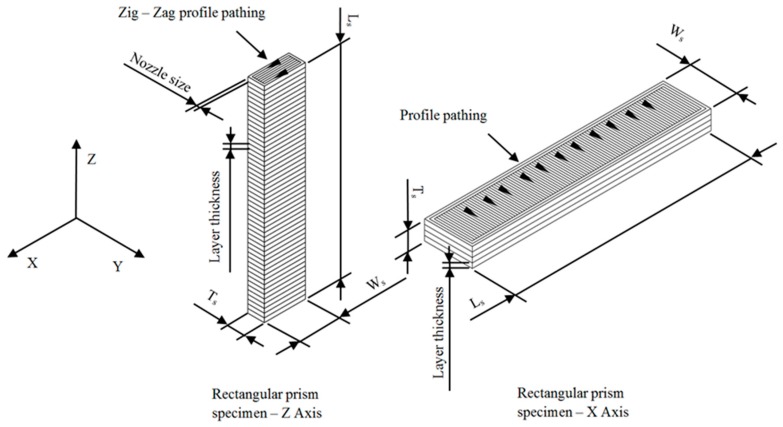
3D printing process arrangement for X and Z axis printed specimens studied in the experimental test.

The characterization of the elastic and mechanical properties of the Nylstrong GF–PA6 3D printing material has been determined from the ISO-604 standard. This standard determines the methodology required to define the elastic and mechanical properties of a plastic subjected to a tensile state uniaxial compression. The experimental tests are carried out by using 3D printing specimens with parameterized prismatic geometry (see Figure 5) and manufactured according to the main directions X and Z in which the material properties have been determined. Table 4 shows the magnitude of the geometric variables used for the calculation of both the uniaxial compression elastic module and the compression yield stress. The magnitude of the manufacturing specifications for producing the specimens along the X and Z directions is shown in Table 3, and these are the same as those employed for manufacturing the end part.

According to the ISO-604 standard used in this manuscript, at least five 3D printing specimens must be tested experimentally for each printing direction in which the mechanical and elastic properties of the plastic material must be obtained. The main directions of analysis of the printed part are X and Z (see Figure 3 and Figure 5), and for this purpose, a total of 11 specimens (five on the X-Axis and six on the Z-Axis) have been manufactured for the experimental tests, all of them having the same dimensions and manufacturing configuration as the end part.

**Figure 6:**
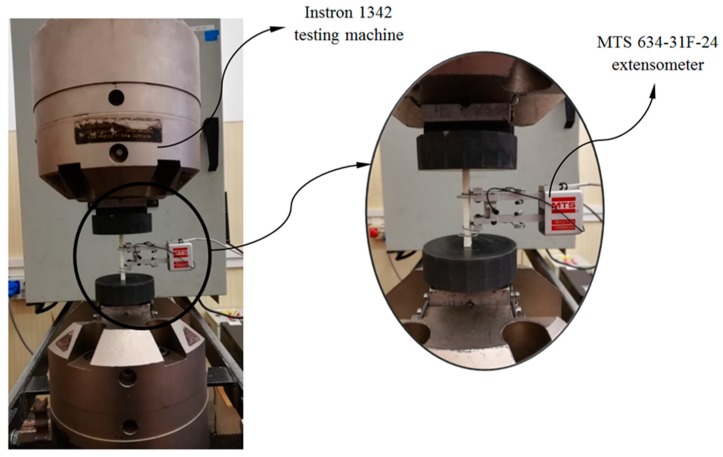
Test of uniaxial compression for specimens, testing machine Isntron 1342 and MTS634-31F-24 extensometer.

Figure 6 shows one of the test specimens located in the compression test machine prior to the experimental test. As is shown, the ends of the specimen are located between the compression machine supports, maintaining the parallelism between both machine supports and the flat supporting surfaces of the specimens. Before starting the experimental test the compression machine makes an adjustment, at low compression speed, of the contact between the supports and the flat contact surfaces of the specimens. In this way is possible to avoid eccentricity and flexo-compression stresses on the specimens during compression tests. Through the experimental test of the specimens the compression speed defined in the machine, from the beginning of the test until the moment of the breaking of the specimens, is constant and equal to 1 mm/min, in line with standard ISO-604 (see Equation (3))

(3) $$v=0.02\times L_{s}$$

where *v* [mm/s] represents the compression speed of the test and *L*_s_ [mm] the length of the specimens tested. In order to obtain the magnitudes of the stress and strain field in the central region of the 3D printing specimens without inertia delay, axial extensometers have been used (see Figure 6). The axial extensometer model used during the experimental uniaxial compression tests on 3D printing specimens is MTS 634-31F-24. On the other hand, the compression machine used to carry out the experimental tests of the 3D printing specimens and the mechanical element under study is the Isntron 1342 (see Figure 6), which complies with the ISO-5893 standards. The test machine Instron 1342 is servohydraulic and includes two supports made of hardened steel and parallels with a plane perpendicular to the compression load axis. In addition, it also incorporates a force transducer mechanism MTS 10 kN, used to record the uniaxial compression load applied to each specimen during the experimental tests. Table 5 and Table 6 show the technical characteristics of the testing machine and the extensometer used for the experimental tests.

**Figure 7:**
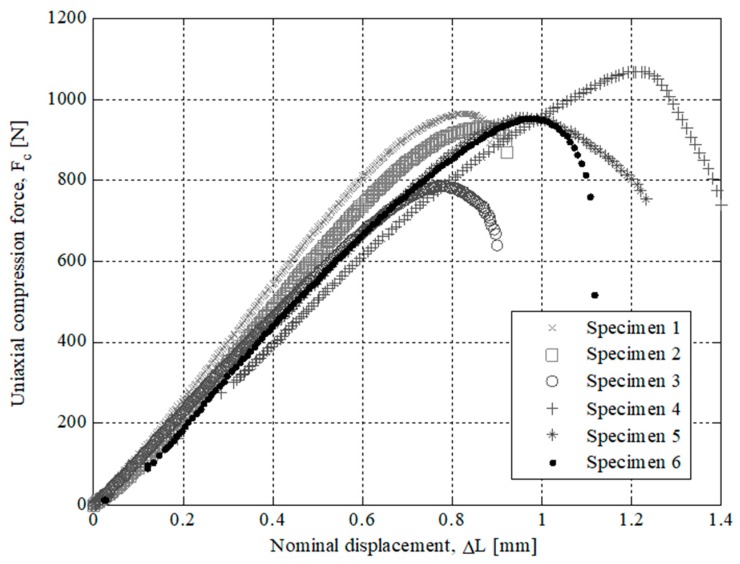
Curves for Z Axis manufactured specimens related to Uniaxial compression force versus nominal displacements.

**Figure 8:**
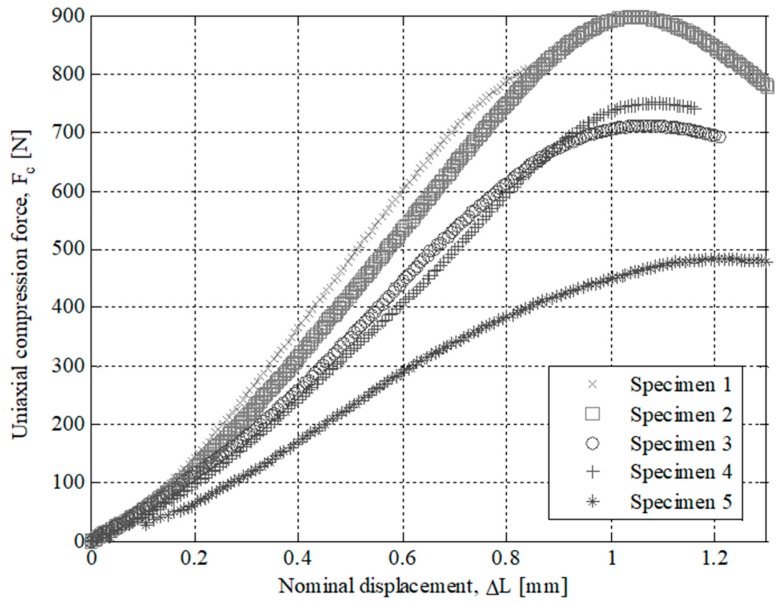
Curves for X-Axis manufactured specimens related to Uniaxial compression force versus nominal displacements.

**Figure 9:**
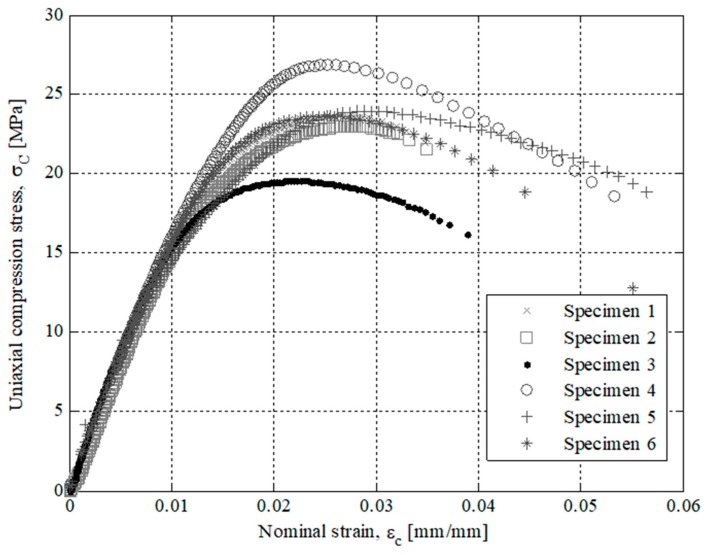
Curves for Z Axis manufactured specimens related to uniaxial compression stress versus nominal strains.

**Figure 10:**
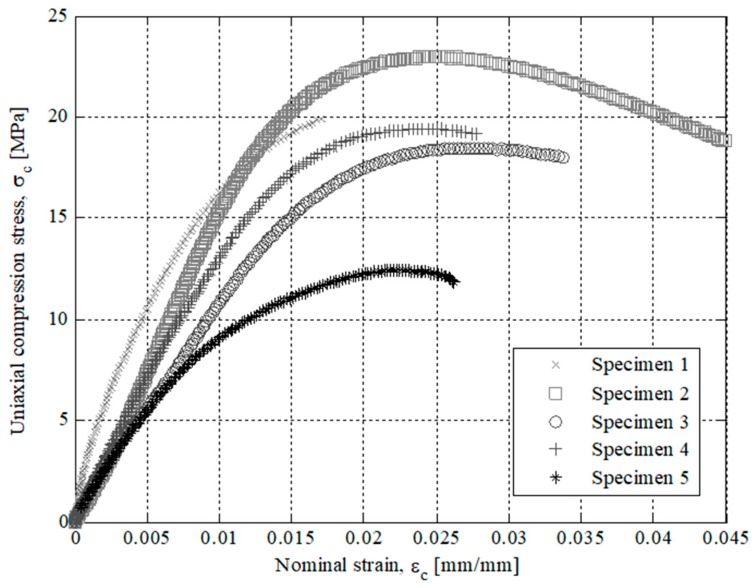
Curves for X-Axis manufactured specimens related to uniaxial compression stress versus nominal strains.

The results of the experimental tests for the specimens tested are presented in Figure 7, Figure 8, Figure 9 and Figure 10. On the one hand, Figure 7 and Figure 8 show the field of uniaxial compression forces and the field of nominal displacements for the 3D printing tested specimens from the beginning of the experimental test until the structural failure of the specimens. The magnitude of these experimental variables is obtained through the transducer mechanism MTS 10 kN included in the testing machine. On the other hand, Figure 9 and Figure 10 show the field of stresses and deformations from the beginning of the experimental test until the structural failure of each specimen. This measurement is taken in the central-cross section of the 3D printing specimens (see Figure 6), which in turn is the most representative area to achieve the elastic and mechanical material features related to the ISO-604 standard. Similarly, Figure 7, Figure 8, Figure 9 and Figure 10 show the results obtained for the specimens printed in the direction of the X and Z axis

As shown in the stress-strain curves (σ_c_-ε_c_), see Figure 9 and Figure 10, the behavior of Nylstrong GF–PA6, used in the manufacture of the X-Axis and Z-Axis tested specimens, is elastic in the initial part of the curves until it reaches the yield strength at a compression value σ_y_ (see Table 7 and Table 8). From this magnitude of stress, the process of non-linear plasticization of the specimens begins until they reach the fracture stress σ_f_. It can be seen that there is a difference in the elastic behavior in the initial part of the stress-strain curve (σ_c_-ε_c_) between the Z-Axis tested specimens and X-Axis tested specimens. On the one hand, Z-Axis tested specimens have a purely linear elastic behavior (see Figure 9), obtaining the elastic module from two pairs of uniaxial compression stress values corresponding to the nominal strain values of 0.0025 and 0.0005 (see Equation (4)), following the requirements of the ISO-604 standard.

(4) $$E_{c}=\frac{\sigma_{c}\left(\varepsilon_{c}=0.0025\right)-\sigma_{c}\left(\varepsilon_{c}=0.0005\right)}{0.0025-0.0005}$$

However, X-Axis tested specimens present a non-linear elastic behavior (see Figure 10) since the evolution of uniaxial compression stresses versus nominal strains is not defined by a line with a constant slope. This non-linear elastic behavior, typical of plastic and foam materials, can be characterized by two elastic parameters: tangent elastic module and secant elastic module. In this way, the tangent elastic modulus is used to characterize stress states of the plastic material in the initial area of the stress-strain curve (σ_c_-ε_c_) while the secant elastic modulus is used to characterize tensile states of the plastic material close to the compressive yield stress σ_y_. Therefore, given that in this manuscript the end part is subjected to a scenario of loads and boundary requirements that cause a stress state of uniaxial compression close to the yield stress σ_y_, the secant module is used as an elastic parameter to characterize the elastic behavior of plastic material for 3D printing with Nylstrong GF–PA6. The magnitude of the secant elastic modulus is established as a γ percentage of the uniaxial compression elastic modulus (see Equation (5)). The value of this percentage γ can vary between 0.70 and 0.85, depending on the type of plastic or foam material and the non-linear elastic region of the stress-strain curve (σ_c_-ε_c_) where the mechanical behavior of the material is analyzed.

(5) $$E_{s}=\gamma E_{c}$$

To determine the elastic compression module of the 3D printing material Nylstrong GF–PA6, both for the specimens manufactured in X-axis and in Z-axis (see Table 7 and Table 8), the arithmetic mean of Young’s modulus compression values obtained for each specimen tested is established. Table 7 and Table 8 show the elastic and mechanical properties for the Z-axis and X-axis of Nylstrong GF–PA6 manufactured with 3D printing.

The characterization of the elastic behavior of Nylstrong GF–PA6 for FDM is carried out according to the guidelines of the analytical model of the ISO-604 standards being used for the definition of the numerical model. In this way, it is possible to validate and compare through mechanical simulations the numerical virtual results with the experimental results obtained from the tests performed on the end part.

The type of fracture that occurs in the X-axis and Z-axis tested specimens is of the fragile type. On the one hand, for the specimens manufactured in the X-axis, the fracture is related to their manufacturing process, since this is mainly caused by complete delamination between adjacent layers of plastic material. On the other hand, for the specimens manufactured in the Z-axis, the fracture is caused by the structural failure of the plastic material, which is to say when the elastic properties limit is exceeded. In both cases, the fracture is carried out in the central cross-sections of the specimens and is produced by the flexural stresses to which the specimens are subjected during their fracture process.

**Figure 11:**
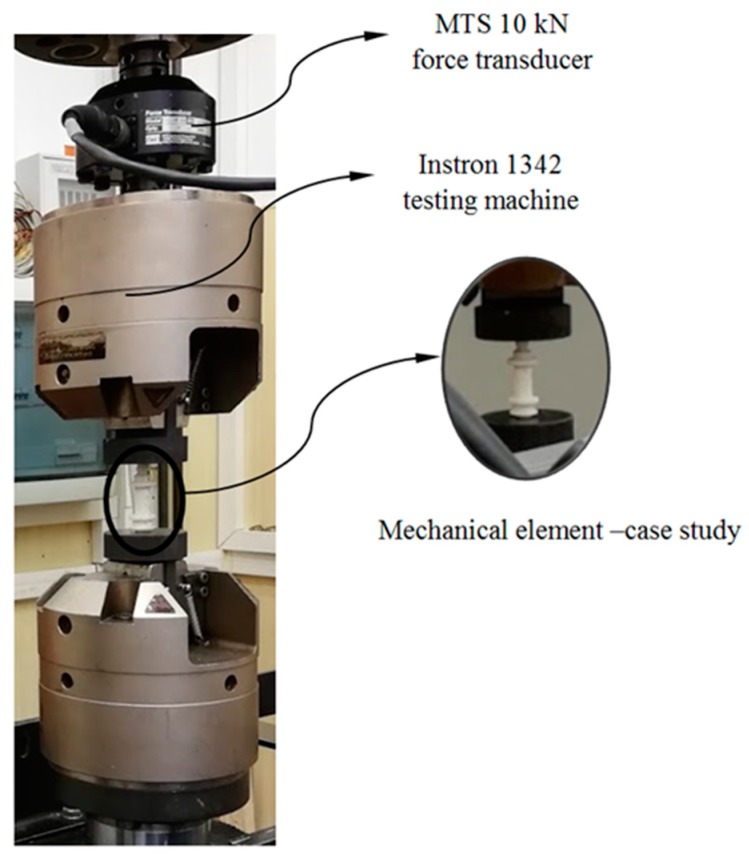
Uniaxial compression experimental test for the printed part under study.

**Figure 12:**
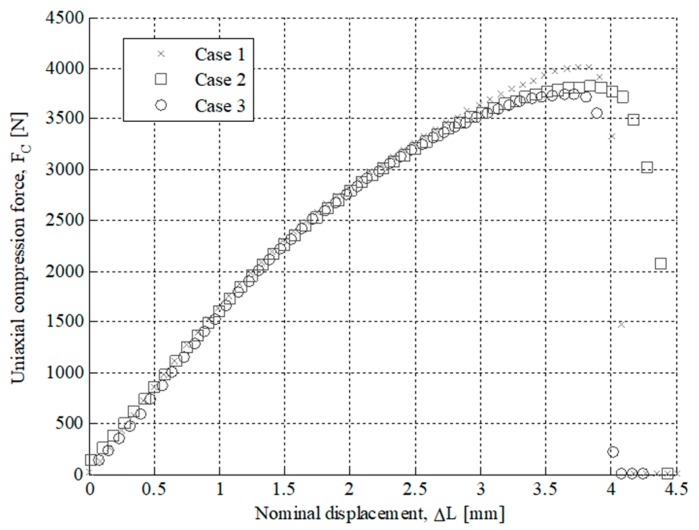
Uniaxial compression load facing nominal displacements for the Z axis printed part tested.

**Figure 13:**
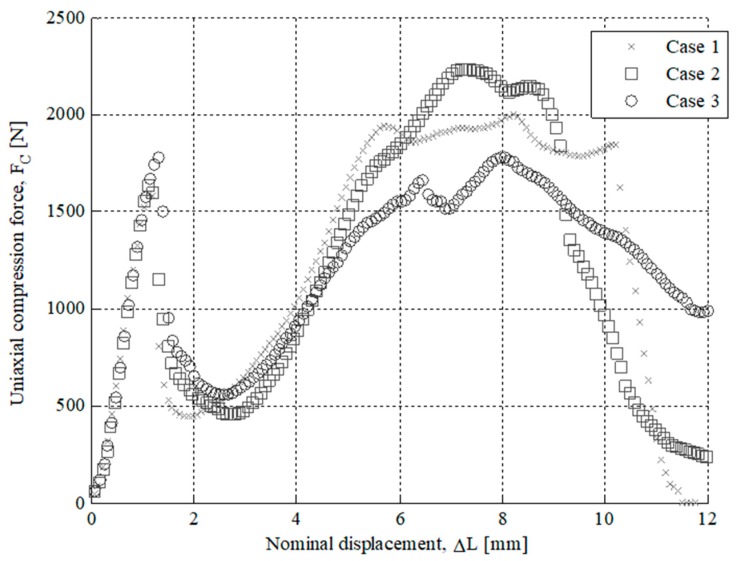
Uniaxial compression load facing nominal displacements for the X-axis printed part tested.

After performing the experimental test with the test specimens, a second experimental test has been carried out in order to analyze the structural behavior for the part under study. The printed end part has been manufactured following the main directions of analysis X and Z and maintaining in both cases the manufacturing parameters in order to evaluate the influence of the manufacturing process on the structural behavior. For both manufacturing directions, three parts have been experimentally tested. In a similar manner to the tests carried out for the 3D printing test specimens, the uniaxial compression test of the end part (see Figure 2) has been performed on the Isntron 1342 test machine (see Figure 11). Figure 12 and Figure 13 show the plots for the uniaxial compression load versus the field of nominal compression displacements to which the end part has been subjected in the compression test. These values were recorded until the state of fracture or structural failure for the tested end parts. Table 9 and Table 10 show the maximum uniaxial compression load and the nominal displacement for the point of fracture for each case study of the end part. According to the curves of uniaxial compression force versus nominal displacement (*F*_c_ − Δ*L*_c_) (see Figure 12 and Figure 13), it can be seen that the fracture in both printing directions differs both in the value of the maximum uniaxial compression force and in the typology of the structural failure.

**Figure 14:**
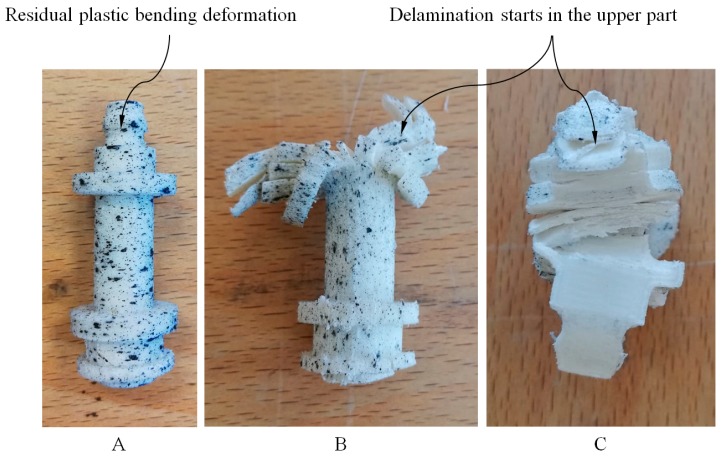
Fracture of the printed part under study: (**A**) Z-Axis; (**B**) X-Axis, front view; (**C**) X-Axis, top view.

As shown in Figure 12 and Figure 14, for the case study of the end part manufactured in the Z-axis the fracture is caused by the residual plastic deformation produced by the bending stresses to which the central region of the mechanical element is subjected. This structural failure collapses the entire geometric domain of the end part since the initial fracture propagates along with the geometry until reaching a maximum compression force of 4018.1 N corresponding to a nominal displacement of 3.746 mm (see Table 9 and Figure 12). On the other hand, as shown in Figure 13 and Figure 14, for the case of the end part manufactured in the X-axis the fracture is generated by delamination in the upper region (see Figure 2) extending towards the rest of its geometric domain. So once the upper region of the end part has collapsed, the magnitude of the uniaxial compression force rises again until the plasticization and structural failure of the central region of its geometric domain. This occurs for a maximum compression force of 1781.5 N corresponding to a nominal displacement of 1.309 mm (see Table 10). In both cases, the type of fracture obtained is of the fragile type because the compressive yield stress is close to the fracture yield stress.

As shown in Figure 12 and Figure 13, the fracture produced during the experimental test for the end part is brittle and occurs due to the structural failure of the material in the circular cross-section where the uniaxial compression force is placed (see Figure 4). On the one hand, for the geometry manufactured in the Z-axis, the structural failure collapses the entire geometric domain of the end part under study, since the initial fracture propagates along the geometry until reaching a maximum compression force of 4018.1 N corresponding a nominal displacement of 3.746 mm (see Table 9 and Figure 12). On the other hand, for the geometry manufactured following the X-axis this structural failure collapses only the geometrical region corresponding to the circular cross-sections of the end of the part where the uniaxial compression force is applied. So once this geometric region has collapsed and plasticized, for a maximum compression force of 1781.5 N corresponding to a nominal displacement of 1.309 mm (see Table 10), plasticization of the central region of the mechanical element begins until its final breakage (see Figure 13).

### 2.3. Fractography

**Figure 15:**
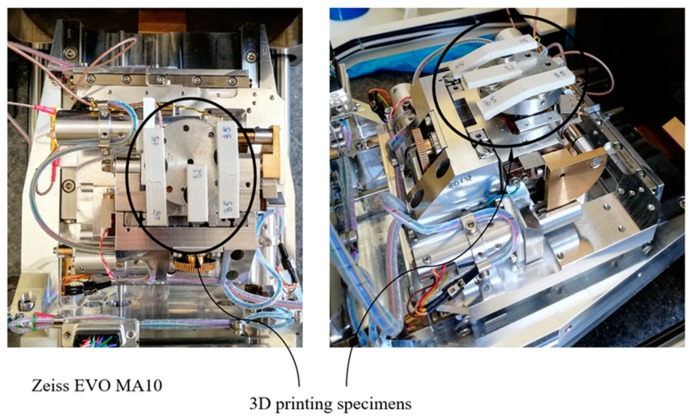
Set–up for the fracture surfaces analysis.

To examine the fracture surfaces of test specimens in the two different directions (X and Z), a scanning electron microscope (SEM, Zeiss EVO MA10 equipped with energy dispersive spectrometer, EDS, Bari, Italy) was used. The failure analysis of polymer specimens reinforced with short glass fibers was carried out by secondary electron imaging using beam energy of 10 kV and a probe current of 1.4 nA. The detailed setup of the SEM is shown in Figure 15.

**Figure 16:**
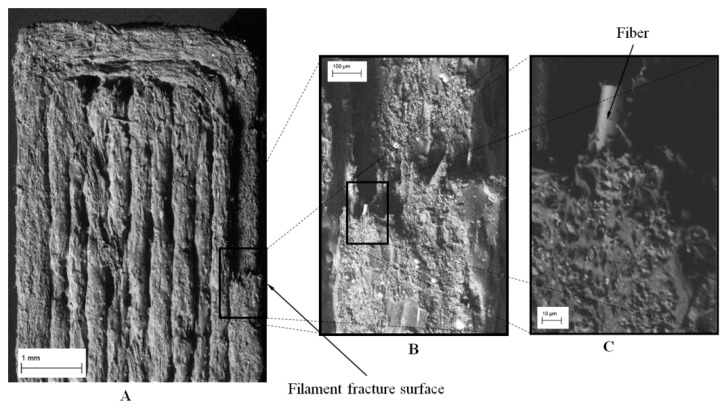
SEM images of Z-direction specimen—top view: (**A**) general view of the surface; (**B**) fracture surface of the contour line; (**C**) fiber rupture.

The specimen’s fracture surface analyses were conducted to examine the fracture mechanism of the fabricated specimens for the two different deposition directions. It can be noticed that the Z-direction specimen (see Figure 16) exhibits an inter-layer brittle fracture. Generally, the fracture is due to complete delamination between two adjacent layers, while the filaments remain intact. However, the fracture growth ends with the breaking of the contour filament along the cross-section (see Figure 16A,B). Also, in this case, the cross-section fracture surface of the filament does not show local plastic effect, but internal fibers were pulled out from the plastic matrix (Figure 16C).

**Figure 17:**
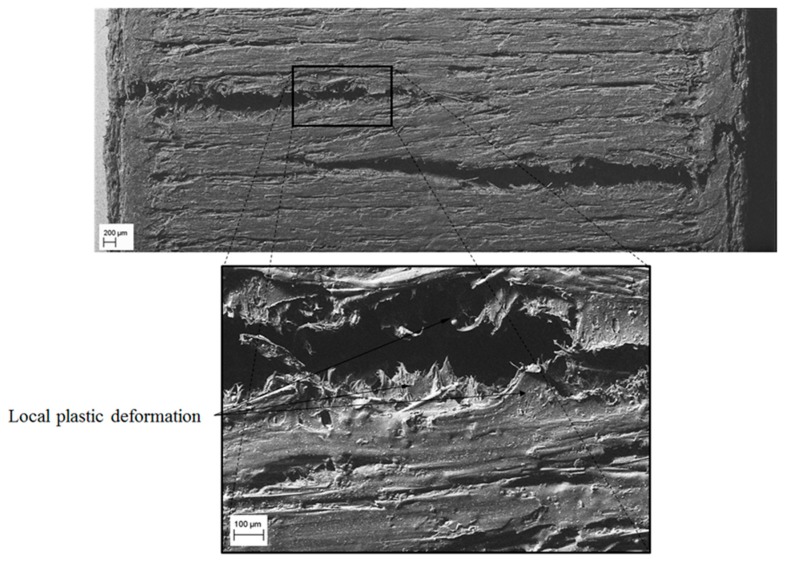
SEM images of the X-direction specimen—front view.

**Figure 18:**
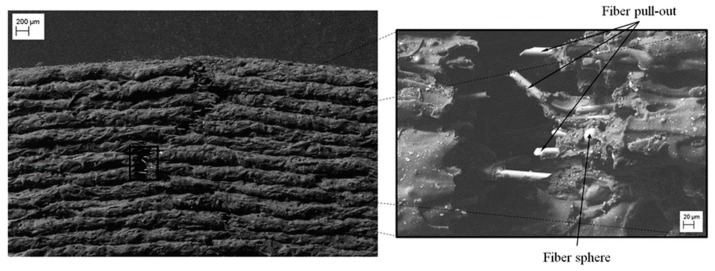
SEM images of X-direction specimen—lateral view.

Figure 17 and Figure 18 show the fracture surface of the X-direction specimen. The front view (see Figure 17) shows that the fracture direction is transverse to the load direction and proceeds from the outside to the inside of the specimen. Furthermore, the edges of the fracture surface show a local plastic deformation of the matrix along the bonding between filaments. The lateral view (see Figure 18) highlights the fact that, due to the flexion load caused by the buckling of the specimen, the crack advances from extrados to the inner part of the specimen. In this configuration, the fracture mode is mainly related to the mechanical behavior of the base material since the filaments bonding direction is perpendicular to the compressive load.

In addition, from Figure 18 it can be noticed the pull-out and failure of the fibers along all the fracture edges. In particular, the pulled-out fibers show an even surface with an angle of 45°, typical of brittle fracture mode. Finally, Figure 18 shows in detail the incorporation of fiberglass spheres presented by the Nylstrong GF–PA6 3D printing plastic material. According to the information provided by the supplier of the material [47], the purpose of these fiberglass spheres is to stabilize the plastic material during its 3D additive manufacturing process and favor its contraction process in order to reduce the defectology associated with the process of manufacture.

### 2.4. Numerical Method

**Figure 19:**
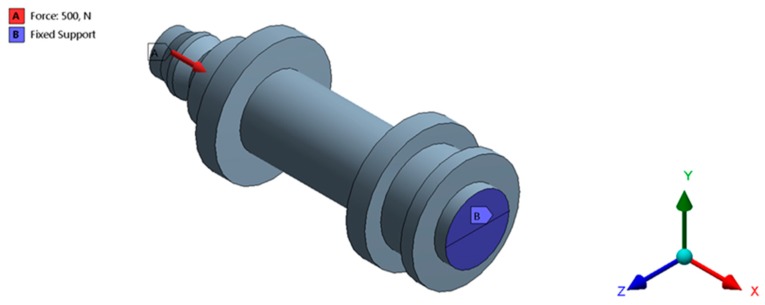
Boundary requirements and loads used for the numerical simulations.

The commercial software used to perform the numerical simulations for the studied part has been Ansys Mechanical [50]. Figure 4 and Figure 19 show the boundary conditions and the load requirements for the numerical part analysis. On the one hand, as loading scenario, a uniaxial compression force is applied along the longitudinal axis of the part at the end to the circular cross-section with diameter Ø_1_ (see Figure 2) and with a value of 500 N. On the other hand, a fixed support is located at the opposite end corresponding to the circular cross-section of diameter Ø_8_ (see Figure 2).

Numerical and mechanical models are established as static and elastic (see Figure 2). Two numerical analyses have been carried out for the part geometry manufactured in the Z and X deposition directions. The objective was first to validate the methodology used for defining the mechanical and elastic properties of Nylstrong GF–PA6 in the numerical software and secondly to compare the numerical and experimental results. For the numerical analysis, the elastic material properties obtained from the experimental test carried out on the specimens both in the Z and X-axis have been employed (see Table 7). For the first numerical analysis, Nylstrong has been defined as isotropic and elastic, with compression Young modulus constant and equal to 960.2 MPa (see Table 7) and a Poisson coefficient of 0.38 (Value obtained from the supplier) [47]. For the second numerical analysis, the elastic properties of Nylstrong obtained from the experimental tests of the specimens manufactured along the X-axis were used (see Table 8). According to the experimental results, it is determined that the elastic behavior of GF–PA6, for the manufacturing direction in the X-axis, is non-linear. Therefore, the material definition for this analysis is established as elastic, isotropic and with a value of the compression secant modulus *E*_*s*_X__ constant and equal to 1083.5 MPa (see Equation (6)). The magnitude of this elastic parameter is determined as a fraction γ of the compressive elastic modulus *E*_*c*_X__ of the plastic material (see Equation (6)). For the manufacturing direction of the X-axis *E*_*c*_X__ is equal to 1354.4 MPa (see Table 8). For the numerical analysis of the printed end part in this manuscript, the γ fraction used to define the magnitude of the secant module is 0.8 (see Equation (6)), according to the criteria established in the ASTM-D695 standard [51]. In this case, the magnitude of the Poisson coefficient is 0.38 (Value obtained from the supplier) [47].

(6) $$E_{s_{X}}=\gamma E_{c_{X}}=0.8\times 1354.4=1083.5\;\mathrm{MPa}$$

**Figure 20:**
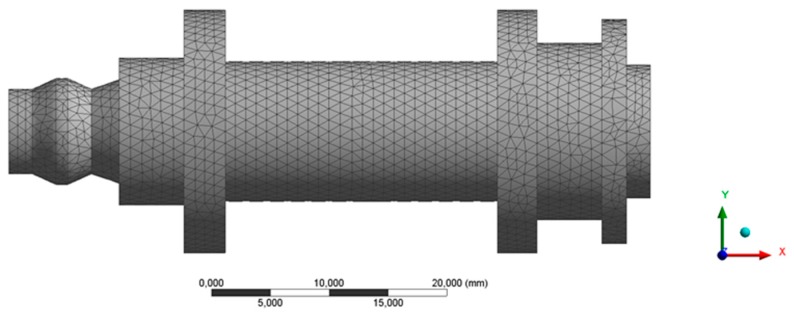
Mesh generated for the numerical simulation.

Solid structural tetrahedral elements SOLID 92 have been employed to discretize the end part topology. Each tetrahedral element is characterized by a quadratic displacement, being composed of 10 nodes where 4 nodes are located in the vertices of the tetrahedron and 6 nodes in the midpoints. Each node has three degrees of freedom including translation in the Nodal X, Y and Z directions. In order to determine the size of the mesh elements, a mesh dimensioning operation is defined, obtaining, as a result, a section size of 1 mm. Table 11 presents the statistics for the numerical simulations performed and Figure 20 shows the mesh. Based on the characterization model of Nylstrong GF–PA6 for the numerical simulation, the large displacement option has been used in the initial solver definition to ensure the convergence of the final solution.

## 3. Results and Discussion

**Figure 21:**
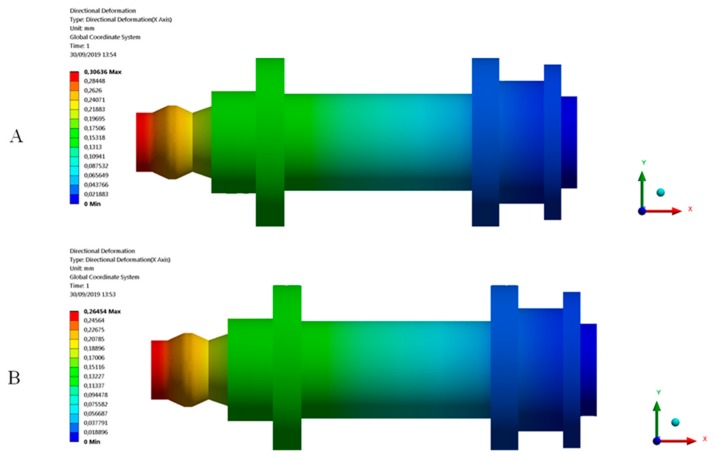
Field of displacements. (**A**) Z-axis printing direction; (**B**) X-axis printing direction.

**Figure 22:**
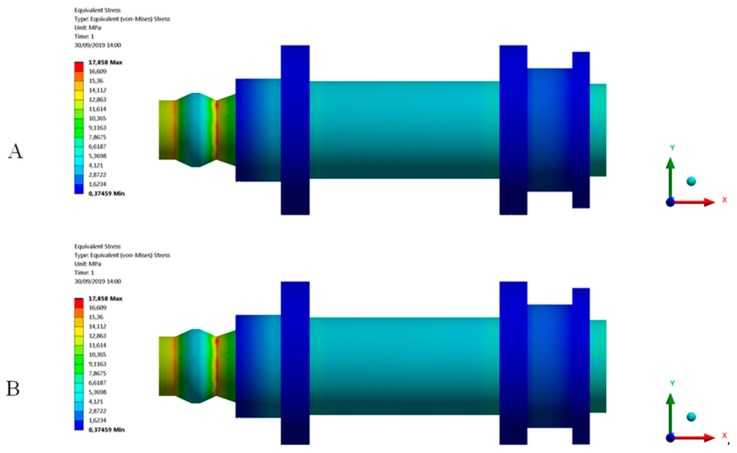
Field of Von-Mises stress obtained in the mechanical simulations: (**A**) Z-axis printing direction; (**B**) X-axis printing direction.

After the experimental evaluation of the structural behavior for the end part subjected to uniaxial compression loads (see Figure 4 and Figure 19) in both the X and Z manufacturing directions (see Figure 3), it is possible to assure that the part structural safety will not be engaged, in any case, for the boundary requirements and the forces to which the part is subjected. The displacement fields resulting from the two numerical simulations both in the Z and X-axis are shown in Figure 21. As shown in Figure 12, Figure 13 and Figure 22, for a uniaxial compression force of 500 N the end part printed in the Z-axis has a nominal displacement of 0.3036 mm and a stress state of 17.858 MPa. On the other hand, for the uniaxial compressions force of 500 N the end part printed in the X-axis has a nominal displacement of 0.2660 mm and a stress state of 17.858 MPa. Thus it can be said that the structural behavior of the end part under the boundary stress conditions to which it is subjected is far from collapse or fracture. The condition of collapse or fracture for the end part is achieved for a uniaxial compression force of 4018.1 N with a nominal displacement of 3.746 mm and 1781.5 N with a nominal displacement of 1.309 mm, for the Z and X axes respectively (see Table 9 and Table 10). These values exceed the stresses to which the end part will be subjected in its load operation. In this sense, it is possible to validate the use of the material Nylstrong GF–PA6, as well as the manufacturing FDM process for the component, studied according to the presented operating requirements.

The maximal value of nominal deformation to characterize the elastic properties of Nylstrong GF–PA6 under compression loads should accomplish Equation (7) according to the ISO-604 standard.

(7) $$\varepsilon_{c}^{*}\leq 0.4\times \frac{T_{s}^{2}}{L_{s}^{2}}\to 0.0025\leq 0.4\times \frac{4^{2}}{50^{2}}=0.0026$$

The geometrical features of the specimens used for characterizing the Nylstrong GF–PA6 material have been presented in Equation (7). Where *T*_s_ [mm] makes reference to the specimen thickness, ε*_c_ to the nominal maximum deformation for obtaining the elastic properties of GF–PA6 and *L*_s_ [mm] to the specimen length. The ISO-604 standard requirements have been fulfilled since the maximum nominal deformation that characterizes the compression Young’s modulus has been 0.0025 less than 0.0026, the deformation value presented in the inequality (see Equation (7)).

The numerical simulation corresponding to the characterization of the GF–PA6 plastic material in the Z manufacturing direction presents a maximum displacement in the direction of load application equal to 0.3064 mm. On the other hand, in the second numerical simulation, where the Nylstrong GF–PA6 plastic material is characterized in the X manufacturing direction, the maximum displacement (in the direction where the load is applied) obtained at the end where the uniaxial compression load is applied is equal to 0.2645 mm. The comparison between the numerical results and experimental ones presents a percentage of relative error of 0.910% (Z-axis) and 0.567% (X-axis), respectively (see Table 12). These results validate the numerical model as an evaluation tool for the Nylstrong GF-PA6 plastic material, as well as for the structural behavior of the printed end part.

Thus, based on the elastic properties obtained from the experimental results according to the ISO 604 standard, it is inferred that the GF–PA6 material manufactured additively with FDM technology can be characterized as isotropic for the structural numerical analysis of parts subjected to uniaxial compression stress states. The numerical modeling of isotropic materials is easy to integrate into FEM analysis software given that it does not need to modify the characterization of the material in the databases. Additionally, it is not necessary to select the correct orientation of the material in the numerical model. However, for the range of work outside the elastic area the material behaves as anisotropic, considering these features in the numerical model of the material.

## 4. Conclusions

The manuscript presents the experimental and numerical study for the mechanical characterization of the Nylstrong GF–PA6 plastic material manufactured using FDM technology in compression uniaxial stress fields. Firstly, an experimental test using several test specimens fabricated with FDM according to the manufacturing directions of the Z and X axes characterized the elastic area of GF–PA6 in both orientations according to the specifications and conditions detailed in the standard ISO 604 for compression stress loads under uniaxial boundary conditions. Secondly, an experimental test analyzed the mechanical characteristics of an end part manufactured in the same conditions as the test specimens, allowing the establishment of compression yield stress, the compression stiffness, stress in the elastic region before the compression yield stress value, displacements and ultimate yield stress parameters. The experimental results in test specimens present a difference in the elastic behavior in the initial part of the stress-strain curve in the Z and X axes. Z-axis printed elements present a purely linear elastic behavior, obtaining the elastic module according to the requirements of the ISO 604 standard. However, X-axis tested specimens presented a nonlinear elastic behavior typical of plastic and foam materials. This elastic region is represented by a line with a non-constant slope. The non-linear elastic behavior of the parts manufactured in X-axis has been characterized by two elastic parameters: a first tangent elastic module for characterizing the stress state of GF–PA6 in the initial area of the stress-strain curve and a second secant elastic module for characterizing tensile states of the printed material close to the value of compressive yield stress σ_y_. The fracture of the test specimens manufactured on the Z axis has been shown on the SEM micrographs, this being brittle mainly due to the separation between filaments in adjacent layers of the specimens. Test specimens manufactured with deposition along the X-axis present a structural failure caused by exceeding the elastic limit of the material. Z axis orientation samples presented lower structural behavior. Numerical validation of the industrial part in both Z and X manufacturing directions and according to the guidelines of the analytical model of the ISO 604 standard has been performed. The simulation results and experimental tests present high precision between numerical and experimental models for the analyzed industrial part, obtaining a relative error of 0.91% on the Z axis and 0.56% on the X-axis. The numerical results together with the experimental ones show that the elastic area for GF–PA6 manufactured using FDM technology can be numerically represented as an isotropic material, by using the elastic specifications from the experimental tests according to ISO standard 604 and on the basis of requirements for product manufacturing. The numerical simulation analysis for the real part in both the experimental test and numerical simulations suggests that the nominal displacements and the compression load are adequate for the boundary requirements and loads applied to the part. On balance, this fact confirms the application of Nylstrong GF–PA6 manufactured with FDM technology for the component studied and for the real boundary conditions presented in this paper. Taking all the above points into consideration, it is possible to characterize GF–PA6 as an isotropic material in the numerical simulation software without modifying the modeling equations of GF–PA6 in the data software. The research results indicate that the FDM process using GF–PA6 is encouraging for producing low volumes of industrial components that work under compression loads or are manufactured directly by the user.

## Figures and Tables

**Table 1:** Mechanical, physical properties and printing FDM characteristics for Nylstrong GF–PA6.

Properties	Units	Value
Density	g/cm^3^	1.580
Notched izod impact	kJ/m^2^	11.520
Tensile strength	MPa	160
Vicat softening temperature	°C	212
Print temperature	°C	255 ± 10
Hot pad	°C	90–100

**Table 2:** Geometrical variables for the 3D printed end part.

Nomenclature	Units	Nominal Value	Nomenclature	Units	Nominal Value
Ø_1_	mm	7.200	*L*_1_	mm	9.350
Ø_2_	mm	12.500	*L*_2_	mm	5.500
Ø_3_	mm	20.600	*L*_3_	mm	3.500
Ø_4_	mm	11.850	*L*_4_	mm	23.100
Ø_5_	mm	20.600	*L*_5_	mm	3.350
Ø_6_	mm	15.000	*L*_6_	mm	5.500
Ø_7_	mm	19.000	*L*_7_	mm	2.100
Ø_8_	mm	11.250	*L*_8_	mm	2.000

**Table 3:** Manufacturing parameters of the FDM process for the 3D printed parts.

Manufacturing Parameters	Value X Direction	Value Z Direction	Units
Layer height	0.2	0.2	mm
Line with	0.4	0.4	mm
Contour lines	2	2	mm
Raster angle	90	90	°
Infill density	100	100	%
Overlap	5	5	%
Infill speed	25	25	mm/s
Wall speed	17	17	mm/s
Support pattern	Cross	Lines	-
Support density	50	40	%
Support Z distance	0.2	0.2	mm
Support X-Y distance	0.3	0.3	mm
Support interface	On	On	-
Nozzle size	0.4	0.4	mm
Infill pattern	Zig-Zag profile	Zig-Zag profile	-
Wall pattern	Contour profile	Contour profile	
Adhesion structure	Raft	Raft	-
Extrusion temperature	255	255	°C
Buildplate temperature	60	60	°C

**Table 4:** Specimen dimensions for the characterization of GF-PA6 under compression loads.

Measurement Parameter	Length, *L*_s_	Width, *W*_s_	Thickness, *T*_s_	Unit
Compression Young modulus	50 ± 2	10.0 ± 0.2	4 ± 0.2	mm
Compression yield stress	50 ± 2	10.0 ± 0.2	4 ± 0.2	mm

**Table 5:** Technical characteristics of the extensometer used in the 3D printing specimen experimental tests.

Model Number	Gage Length [mm]	Measuring Range: Strain [mm]
634-31F-24	10–50	+4/−2

**Table 6:** Technical characteristics of the test machine.

Model Number	Cross Head Movement [mm]	Maximum Dynamic Load [kN]	Maximum Static Load [kN]	Strain Gauge [mm]
Isntron 1342	±50	100	200	±4

**Table 7:** Elastic and mechanical properties of Nylstrong GF–PA6 for each Z-axis specimen.

Properties of Compression	Units	S1	S2	S3	S4	S5	S6	Arithmetic Average	Typical Deviation
Young’s modulus, *E*_c_	MPa	974.2	968.5	754.6	1192	834.8	1037	960.2	153.6
Yield stress, σ_y_	MPa	14.2	16.3	17.1	23.1	20.3	20.2	18.5	3.2
Fracture stress, σ_f_	MPa	23.6	22.9	19.5	26.8	23.9	23.6	23.4	2.3

**Table 8:** Elastic and mechanical properties of Nylstrong GF–PA6 for each X-axis specimen.

Properties of Compression	Units	S1	S2	S3	S4	S5	Arithmetic Average	Typical Deviation
Young’s modulus, *E*_c_	MPa	1812	1532	1085	1357	986.1	1354.4	335.1
Yield stress, σ_y_	MPa	19.1	18.8	16.2	17.4	14.2	17.1	2.0
Fracture stress, σ_f_	MPa	20.9	23.0	18.5	19.4	15.4	19.4	2.8

**Table 9:** Mechanical properties for the printed part achieved in the uniaxial compression test in Z-axis.

Properties of Compression	Units	Value
Uniaxial maximum force	N	4018.1
Nominal displacement at maximum uniaxial force	mm	3.746

**Table 10:** Mechanical properties for the printed end part achieved in the uniaxial compression test in X-axis.

Properties of Compression	Units	Value
Uniaxial maximum force	N	1781.5
Nominal displacement at maximum uniaxial force	mm	1.309

**Table 11:** Statistics of the mesh generated for the mechanical modeling.

**Number of Elements**	68,678
**Nodes Number**	100,125
**Quality of the Element (Average)**	0.841

**Table 12:** Comparison of the experimental and numerical results obtained on the end part under the load scenario to which it is subjected.

3D Printing Direction	Experimental Displacement [mm]	Numerical Displacement [mm]	Relative Error [%]
Mechanical element-Z	0.3036	0.3064	0.910
Mechanical element-X	0.2660	0.2645	0.567
